# Volumetric absorptive microsampling to measure iohexol and creatinine concentrations for estimation of glomerular filtration rate in cats: aligning animal welfare with practical feasibility

**DOI:** 10.1186/s12917-025-04748-2

**Published:** 2025-04-27

**Authors:** Hanna De Baets, Marleen Brans, Dominique Paepe, Christophe P. Stove

**Affiliations:** 1 Laboratory of Toxicology, Department of Bioanalysis, Faculty of Pharmaceutical Sciences, Ghent University, Ghent, Belgium; 2 Small Animal Department, Faculty of Veterinary Medicine, Ghent University, Merelbeke, Belgium

**Keywords:** Liquid chromatography-tandem mass spectrometry, Volumetric absorptive microsampling, Glomerular filtration rate, Chronic kidney disease, Creatinine, Iohexol

## Abstract

**Background:**

Chronic kidney disease (CKD) is common in cats, and early detection is crucial for better prognosis. Currently, the gold standard to assess renal function is the measurement of glomerular filtration rate (GFR), allowing early detection of decreased kidney function. To overcome the practical limitations of this procedure, microsampling, collecting a small drop of blood from the cat’s ear, can be used. Application of volumetric absorptive microsampling (VAMS) in feline nephrology would be of tremendous value, aligning with animal welfare and improving practical feasibility of GFR measurements.

**Results:**

We developed and successfully validated liquid chromatography – tandem mass spectrometry (LC-MS/MS) methods to simultaneously determine iohexol and creatinine in plasma, blood and VAMS samples. A clinical validation study, conducted in 23 cats from whom conventional venous blood, plasma and VAMS samples were collected, allowed to establish a conversion formula to derive plasma iohexol or creatinine concentrations from capillary VAMS concentrations. This conversion was applied on an independent set, revealing an excellent agreement for both iohexol and creatinine between concentrations directly measured in venous plasma or derived from ear-prick VAMS samples (94% and 96% of differences lay < 20%, respectively).

**Conclusions:**

We demonstrated that ear-prick sampling using VAMS is a suitable alternative to conventional venous sampling to measure iohexol and creatinine for GFR determination in cats.

**Supplementary Information:**

The online version contains supplementary material available at 10.1186/s12917-025-04748-2.

## Background

Chronic kidney disease (CKD) is one of the most common diseases in cats and accounts for substantial mortality, especially in older animals [1]. Currently, routine diagnosis of CKD is mainly limited to the detection of advanced azotemic disease (increased serum creatinine and/or urea in combination with decreased urine concentrating ability) [2]. Because these biomarkers only begin to show abnormalities in an advanced disease stage, they are insensitive markers for early diagnosis of renal failure [3, 4]. Recently, also symmetric dimethylarginine (SDMA) titration has emerged as a more sensitive marker for detection and monitoring of CKD, although it can be influenced by other factors than CKD and must hence be interpreted alongside other diagnostic markers. Importantly, early detection of CKD is crucial for improving the prognosis of the cat. Because timely therapeutic intervention can prevent or delay disease progression and complications, survival time increases as the animal is diagnosed in earlier stages of the disease (International Renal Interest Society (IRIS) Stages) [2, 3, 5].

The most sensitive and accurate measure to assess kidney function is the evaluation of the glomerular filtration rate (GFR) [4]. GFR measurement allows early detection of decreased kidney function (IRIS stage I and early stage II), before azotemia develops (advanced IRIS stage II or higher) [6]. However, the gold standard method to measure GFR (urine clearance of inulin) has important practical limitations and is not used in clinical practice [4]. Recently, iohexol has been suggested as an ideal exogenous filtration marker and has been shown to provide reliable GFR assessments in animals as well as in humans [7–9]. In cats, both iohexol and exogenous creatinine are used in plasma clearance tests and both filtration markers allow reliable estimations of GFR [10]. Still, there is an urgent need for more practical, cost-effective and accurate methods to detect early feline CKD [3].

For GFR evaluation based on plasma iohexol or exogenous creatinine clearance, repeated blood samplings at multiple time points are proposed [8]. However, the practical use of such multi-sampling techniques in cats is hampered because they are time-consuming, labor-intensive and may be painful or stressful for the patient [3]. To reduce the invasiveness associated with conventional blood sampling and to overcome practical hurdles linked to repeated sampling, human medicine has gained interest in microsampling techniques. Microsampling techniques only require a minimal amount of capillary blood (< 50 µL), collected via a finger stick or ear-prick (in cats). Moreover, microsampling devices allow for remote blood sampling and increased analyte stability, while reducing logistic costs for sample transportation [11, 12]. Dried blood spots (DBS) are the best known and most frequently used dried blood microsamples and have been used successfully to measure iohexol-based GFR in human medicine [13–16]. To overcome DBS-related issues such as the well-known hematocrit (hct) effect, more specialized devices such as volumetric absorptive microsampling (VAMS) systems, allowing the collection of a fixed amount of blood, independent of the hct, have been developed [17, 18]. VAMS devices consist of a plastic handler to which a hydrophilic polymeric tip is attached that will wick up an exact volume of e.g. 10 µL of blood, irrespective of the hematocrit. Recently, also methods for the determination of iohexol in VAMS samples have been developed for use in human medicine [19–21].

The application of microsampling has great potential in feline nephrology, not only by enhancing the practicality of GFR procedures, but also by allowing follow-up of the kidney function of cats diagnosed with CKD (via measurement of endogenous creatinine) while supporting animal welfare through its minimally invasive nature. In preclinical animal research, microsampling is a powerful tool that supports the **3R** (reduce, refine, replace) principle by enhancing ethical standards in animal research [22]. The collection of smaller sample volumes allows for multiple samples to be taken from the same animal, dramatically **reducing** the number of required animals in animal experimentation studies. Moreover, by **refining** the procedures, stress and pain for the animals is minimized, while data quality and research efficiency maximize. Consequently, more invasive animal studies can be **replaced** or avoided [11, 23].

In this study, we investigated the use of VAMS-assisted ear-prick sampling as a tool to measure iohexol and creatinine concentrations in cat samples. We developed and analytically validated LC-MS/MS methods for the simultaneous quantification of iohexol and creatinine in blood, plasma and VAMS samples. Particular emphasis was placed on the ethical and sustainable aspects of the method by employing human blood-based matrices for calibration and utilizing microsampling to minimize the reliance on cat blood. As such, the framework of this study may serve as a basis for implementation of the 3R principle in bio-analytical method validation. Subsequent clinical validation was performed by comparing results obtained in plasma, venous (liquid and dried) blood samples and dried capillary blood samples from cats to determine whether capillary ear-prick samples could be used as an alternative matrix to reliably measure iohexol and creatinine concentrations. To the authors’ knowledge, this is the first study (i) applying VAMS technology as such in cats, (ii) comparing all different blood-based matrices for iohexol and creatinine in a time-dependent manner and (iii) providing a successful application of VAMS technology in the context of quantification of iohexol and creatinine concentrations, a vital step to obtain reliable GFR estimations.

## Methods

### Analytical method development and validation

#### Chemicals and materials

Iohexol and creatinine-d3 powder were purchased from Sigma-Aldrich (Diegem, Belgium). Creatinine reference material (SRM 914a) was used from the National Institute of Standards and Technology (NIST). Creatinine-^13^C3d3 was purchased from Alsachim (Illkirch Graffenstaden, France), while iohexol-d5 was from TRC (Toronto, Canada). Creatinine-^13^C was obtained from Cambridge Isotope Laboratories (CIL, Tewksbury, Massachusetts, USA). Formic acid (FA) and LC-MS grade methanol were purchased from Chem-Lab (Zedelgem, Belgium). LC-MS grade acetonitrile (ACN) was obtained from Biosolve (Valkenswaard, The Netherlands). Ultrapure water was produced by a Millipore purification system (Merck Millipore, Overijse, Belgium). LC-MS grade 2-propanol and desiccant packages (10 g Minipax absorbent packets) were obtained from Sigma-Aldrich. VAMS devices (10 µL; brand name Mitra™) were purchased from Trajan Scientific (Victoria, Australia).

#### Standard solutions, calibrators and quality control samples (QCs)

For the internal standards (IS), 5 mg of creatinine-^13^C3d3 and 10 mg of iohexol-d5 were dissolved in ultrapure water and methanol to yield concentrations of 5 and 10 mg/mL, respectively. The IS solutions were stored at -20 °C. On the day of analysis, these were further diluted in 90/10 water/MeOH to generate a solution of 5 µg/mL for creatinine-^13^C3d3 and 5 µg/mL for iohexol-d5.

Stock solutions from creatinine-d3 and iohexol were prepared in water at a concentration of 20 mg/mL. All stock solutions were stored at -80 °C. For the calibrators, a mix of the stock solutions of both analytes was diluted with water to a concentration of 10 mg/mL. This stock solution was diluted to end up with 8 distinct working solutions, each leading to the generation of a single calibrator level. Blood and plasma were spiked with these working solutions, with the spiked volume not exceeding 5% of the total volume. Eight calibrators were prepared for both whole blood and plasma within the calibration range of 2 to 400 µg/mL for both iohexol and creatinine-d3.

In parallel, four QC working solutions were independently prepared from the working solutions for the calibrators to prepare QCs at 4 different levels in human blood and plasma: lower limit of quantification (LLOQ), low (QCL), mid (QCM) and high (QCH) level. Additionally, QCs were also prepared in cat blood and plasma at 2 different levels: QCL and QCH.

The spiked whole blood and plasma calibrators and QCs were briefly homogenized via inverting the 2 mL cups and placing them on a roller to incubate for 30 min at room temperature (RT). Next, calibrators and QCs were generated via dipping the tip of the VAMS device in the blood. These tips consist of a hydrophilic polymer that will wick up an exact volume (here 10 µL) of blood, irrespective of the hematocrit. Once the tip was completely filled, it was held in the blood for an additional 2 s to ensure complete filling and the resulting VAMS samples were dried for at least 2 h under ambient conditions prior to extraction. If samples were not immediately analyzed, they were stored in zip-lock bags containing desiccant and stored at -80 °C until analysis. Also for the analysis of whole blood and plasma, the abovementioned calibrators and QCs were used.

#### Sample collection

The use of blood from healthy, adult, human volunteers was approved by the Ghent University Hospital Ethics Committee (EC-BC 07324) and included a statement of informed consent. Blank venous whole blood, collected in EDTA tubes (BD Vacutainer, New Jersey, USA) and plasma derived thereof was collected from a healthy, female, human volunteer to generate calibrators and QCs. Similarly, cat blood and plasma were collected for the generation of QCs. The use of cat blood was approved by the owners of the animals and included a statement of informed consent. For the evaluation of matrix effects (ME) and recovery (RE), blank EDTA whole blood and plasma derived thereof was collected from 6 healthy, human volunteers. For the generation of VAMS samples with a broad hct range, the blood was centrifuged for 5 min at 4000 g and a specific amount of plasma was added or removed. The plasma was generated by centrifuging an aliquot of whole blood in an Eppendorf 5804R Centrifuge (Hamburg, Germany) for 5 min at 4000 g. A Sysmex XN-1000 hematology analyzer (Sysmex Corp., Kobe, Japan) was used to determine the hct value of the whole blood, whenever necessary.

#### Sample preparation

From the blood and plasma samples, 50 µL was extracted by adding 950 µL extraction solvent containing IS (90/10 water/MeOH). The 2 mL cups were vortexed, followed by 3 min shaking on a Thermoshaker (Biosan, Riga, Latvia) at 1400 rpm and at 24 °C. Next, after vortex mixing, 100 µL was transferred to a 1 mL 96-well (round-well, round bottom) plate (Agilent, Diegem, Belgium) to which 300 µL cold MeOH (stored at 4 °C) was added for protein precipitation. After shaking on a Thermoshaker for 3 min at 1400 rpm and at 25 °C, and subsequent centrifugation for 10 min at 2250 g and at 4 °C, 20 µL of the supernatant was diluted with 100 µL of mobile phase (MP) A (0.1% FA in water) in a 350 µL 96-well sample collection plate (Waters, Milford, MA, USA). This final plate was then centrifuged prior to injection onto the UPLC^®^ system.

For the analysis of the VAMS samples, the tip of the device was detached manually from the handle into a 1 mL 96-well (round-well, round bottom) plate. For the following steps, an automated extraction protocol was developed using the Waters Andrew + pipetting robot. After dispensing 200 µL of extraction solvent with IS (90/10 water/MeOH), the plate was shaken 2 times for 10 min at 1400 rpm and at 24 °C. After addition of 600 µL of 75/25 ACN/MeOH for protein precipitation, the plate was removed manually from the robot and centrifuged 2 times for 5 min at 2250 g and at 4 °C, while using an exact counterweight in order to ensure full precipitation of all proteins (as outlined below). In the meantime, 100 µL of MP A was added to a 350 µL 96-well sample collection plate by the robot. After putting the precipitation plate back in the robot manually, 20 µL of supernatant was transferred to the final collection plate, already containing MP A. This final plate was then centrifuged prior to injection onto the UPLC^®^ system.

#### LC-MS/MS method

All samples were analyzed using a Waters Acquity UPLC^®^ coupled to a Waters XEVO TQ-S mass spectrometer. The hardware was controlled by Masslynx software (version 4.2). Creatinine and iohexol were chromatographically separated on an Acquity UPLC^®^ HSS T3 column (100 × 2.1 mm; 1.8 μm; Waters) with corresponding VanGuard precolumn held at 30 °C. The autosampler was maintained at 10 °C. A 3 min gradient elution program was used with MP A consisting of 0.1% FA in water and MP B consisting of 0.1% FA in MeOH. The flow rate was always set at 0.4 mL/min. The gradient starts at 100% MP A and is steeply increased to 60% MP B after 0.5 min, followed by another steep increase to 100% MP B after 1.1 min, followed by a short isocratic period of 100% MP B for 0.4 min and finally reversal to starting conditions, maintained for 1.5 min, resulting in a total run time of 3 min. The MS was equipped with an electrospray (ESI) source operating in positive ionization mode. An optimized multiple reaction monitoring (MRM) method was used to detect the compounds. The optimized MS parameters were the following: capillary voltage 4.0 kV; desolvation gas (N_2_) temperature 250 °C at a gas flow of 600 L/h; cone gas (N_2_) flow rate 150 L/h with a source temperature of 150 °C; nebulizer gas pressure (N_2_) 7 bar. As collision gas, argon was used at a flow rate of 0.15 mL/min. For all analytes, two characteristic precursor-to-product ion transitions were monitored, while for the corresponding IS one transition was analyzed. MRM transitions, as well as the compound-specific MS parameters, are listed in Supplementary Table 1.

#### Creatinine and creatinine-d3 equivalence

Because of the endogenous presence of creatinine in all native whole blood and plasma samples, creatinine in calibrators and QC samples was substituted by creatinine-d3 as a “surrogate” analyte. In this way, standard addition could be avoided to quantify creatinine. However, we observed a difference in ionization efficiency for creatinine compared to creatinine-d3, resulting in a different abundance of the corresponding *m/z* fragments. Therefore, to ensure a correct quantification of creatinine using this approach, 29 plasma samples were analyzed using creatinine-d3 calibration as well as using a creatinine reference measurement procedure. The method is contained in list I (Reference Measurement Procedures; NRMeth 1) of the Joint Committee for Traceability in Laboratory Medicine (JCTLM) database (see www.bipm.org). Six plasma samples were derived from healthy, human donors and twenty-three plasma samples from cats (both with normal and impaired kidney function). In order to cover the entire calibration range, ten samples were spiked with an additional amount of creatinine (40, 100, 200 or 300 µg/mL).

Based on the agreement between the results of both methods for all 29 creatinine samples, the need for a conversion factor, caused by the use of creatinine-d3 calibration, was evaluated. Passing-Bablok regression analysis was performed to evaluate the presence of systematic and proportional differences.

#### Method validation

The methods were validated based on the European Medicines Agency (EMA) and U.S. Food and Drug Administration (FDA) guidelines on bio-analytical method validation, as well as the IATDMCT guideline for the validation of dried blood spot-based methods [24–26].

The calibration model for the analytes in each matrix was statistically evaluated using an R-script developed by Desharnais et al. [27]. In short, using this script, heteroscedasticity was evaluated via an F-test. Next, a variance test for weight selection was done to select the most appropriate weighting factor. Additionally, a partial F-test was run to determine the model order. Finally, a Cramer von Mises test was performed to verify a normal distribution of the standardized residuals. The % bias of the back-calculations of the calibrators with the selected model should be less than 15% (20% at LLOQ level) for 75% of all calibrators.

Accuracy and precision were evaluated in human blood, plasma and VAMS samples based on the analysis of four different QC levels (LLOQ, QCL, QCM and QCH) over 3 different days, analyzed in duplicate on each day for all matrices. In parallel, accuracy and precision were evaluated in cat blood, plasma and VAMS samples by the analysis of two different QC levels (QCL cat and QCH cat), prepared in blood and plasma from 4 cats, over 3 different days, analyzed in duplicate on each day for all matrices. The acceptance limit for accuracy (% bias) was set at 15% (20% at LLOQ level). Repeatability and total precision were calculated via one-way ANOVA. The % coefficient of variation (CV) limit for the repeatability and total precision was set at 15% (20% at LLOQ level).

Two blank samples (without analyte or IS in the extraction solvent) were injected after the highest calibrator to evaluate carry-over. The response at the retention time of the analyte or IS in the blank samples was considered acceptable if the signal was less than 20% of the analyte peak area at LLOQ and less than 5% of the peak area of the IS.

To assess selectivity, the pre-dosing samples (cat samples) from the clinical validation study were used (as outlined below). For iohexol, the selectivity of the method was confirmed when the signal was less than 20% of the LLOQ area and less than 5% of the IS area. For creatinine, ion ratios were compared between neat standard solutions on the one hand and the native blood, plasma and VAMS study samples on the other hand. The absence of interferences was accepted when the ion ratio of the native samples was within ± 20% of the ratio in the neat standard solutions [28].

ME and RE were evaluated following the approach described by Matuszewski et al. [29]. For this, blank samples from 6 individual human donors were used at two different concentration levels (QCL and QCH). For the VAMS samples, also samples prepared from blood with low and high hct were included (hct of 0.168 and 0.543 L/L). A first set of samples was spiked before extraction (A), a second set after extraction (B), and a third set in the absence of the matrix to be assessed (C). The absolute ME was calculated for each donor by dividing the analyte peak area in the presence of matrix spiked after extraction (B) by the peak area in the absence of that same matrix (C). The relative IS corrected ME (CV) should be below 15%. For the evaluation of the RE, the signals obtained in samples spiked before (A) and after (B) extraction were compared. For RE, the relative RE (CV) should be below 15%.

For the VAMS samples, in addition to evaluation of the RE at normal hct (0.35 L/L), a possible impact of the hct on the RE was also evaluated at five different hct levels (target values at 0.17, 0.25, 0.35, 0.45 and 0.55 L/L). Human blood at all hct levels was spiked with the analytes at Low and High QC level before and after extraction of the VAMS samples (*n* = 6 for each condition). The RE was calculated by dividing the peak areas at each condition in the set spiked before extraction by those of the corresponding samples spiked after extraction. The RE for the lower and higher hct blood were compared to the RE at hct 0.35 L/L (considered the reference hct). The relative IS-compensated RE for the lower and higher hct levels should be within ± 15% of the RE for VAMS samples prepared from blood with a hct of 0.35 L/L to be considered hct-independent.

Because the stability of iohexol and creatinine has already been investigated in blood, plasma and dried blood samples [8, 21, 30], stability was only investigated at the storage conditions applied to the actual study samples. For blood and plasma, stability was evaluated after storage for 1 week at 4 °C for QCL, QCH and two native cat samples (‘cat sample 1’ and ‘cat sample 2’, containing iohexol and creatinine) (*n* = 3). For the VAMS samples, stability was evaluated after storage for 1 week at RT and for 2 days at 60 °C for QCL, QCH and two native cat samples (‘cat sample 1’ and ‘cat sample 2’, containing iohexol and creatinine) (*n* = 3). For storage, the VAMS samples were put into zip-lock bags containing desiccant. For all evaluations, stability studies were performed isochronally. All samples were analyzed together within one run, and the analytes were considered stable under the specific storage conditions when the deviation was less than 15% compared to the concentration determined from the reference samples (t_0_ samples which were stored at -80 °C during the entire duration of the stability studies).

For the evaluation of long term stability at -80 °C, cat blood and plasma samples collected during the GFR protocol from 2 cats were pooled to obtain a ‘pool’ with a relatively low and relatively high concentration of both analytes. Blood, plasma and VAMS samples (generated from the whole blood ‘pools’) were stored at -80 °C and analyzed in triplicate on each study sample analysis day. In this way, the stability could be assessed longitudinally and the analytes were considered stable in the different matrices if the deviation was within 15% of the concentration determined at the first study sample analysis day (t_0_).

Stability of the extracts for all matrices was also evaluated at low and high QC level, in addition to four cat samples (QCL and QCH samples from 2 cats). Sample extracts (*n* = 2) were reinjected after 24 and 48 h in the autosampler, held at 10 °C, and after 2 weeks at -20 °C (one freeze thaw cycle), together with the calibration curve that had been stored under the same conditions. The % deviation from time 0 (t_0_) was evaluated and should be less than 15%.

### Clinical method validation

#### Study design

After the bioanalytical method validation, a clinical validation study was performed for iohexol and creatinine. Client-owned cats and cats with (early and advanced) CKD were selected from an ongoing prospective study approved by the Ethics Committee of the Faculty of Veterinary Medicine and Faculty of Bioscience Engineering (Ghent University) (EC 2021/075). Approval by the owner was confirmed with a signed informed consent. Further details on the included cats can be found in Supplementary Table 2.

#### Sample collection and analysis

**Fig. 1:**
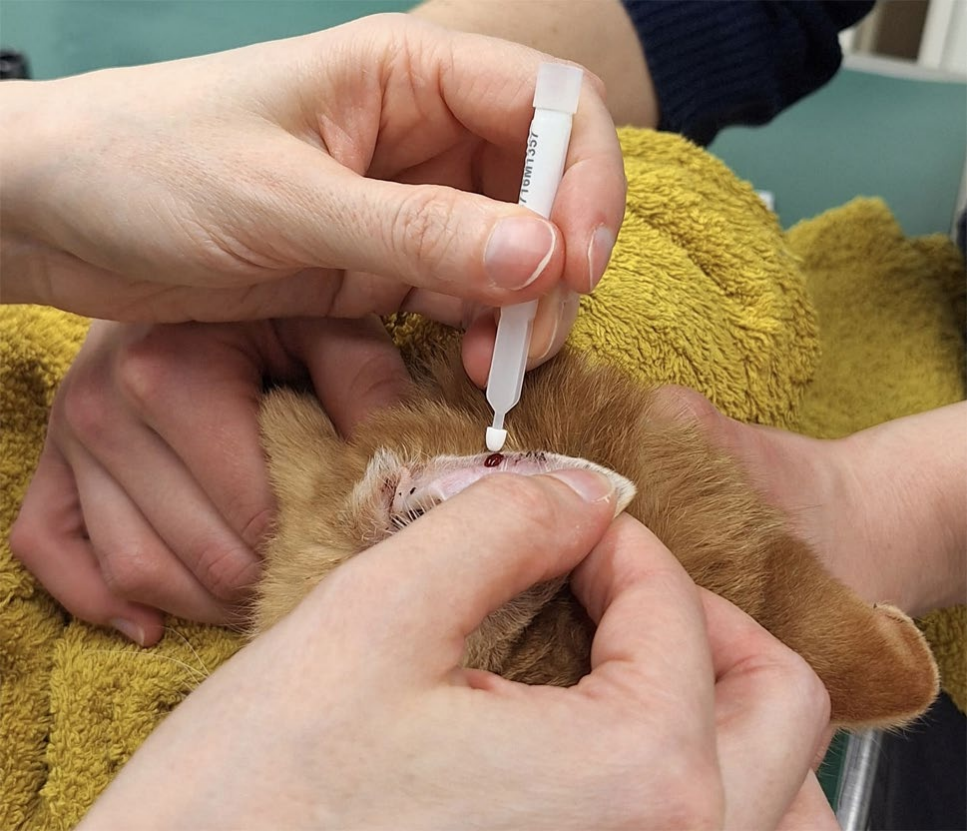
Collection of a VAMS sample from a cat included in the study after performing an ear-prick. VAMS *volumetric absorptive microsampling*

Venous EDTA-anticoagulated blood (max. 1 mL per collection time point) (collected in 4.0 mL BD Vacutainer tubes; BD Benelux, Erembodegem, Belgium) and duplicate 10 µL capillary VAMS (cVAMS) samples obtained after an ear-prick were collected from 23 cats before and after they received a single intravenous bolus injection of iohexol (Omnipaque, 64.7 mg/kg body weight) and creatinine (40 mg/kg body weight). No sedation was performed prior to sample collection. A photograph of the collection of the cVAMS sample is included in Fig. 1. During the GFR protocol, samples were collected before administration (t_0_, pre-dosing samples) and 5, 30, 60, 120, 180, 360 and 600 min after administration of iohexol and creatinine. Additionally, plasma was derived from the venous blood, and duplicate venous VAMS (vVAMS) samples were prepared from the venous blood within 1 h after collection. For the collection of the cVAMS samples, the ear was cleaned with an alcohol wipe, dried, and punctured using a contact-activated safety lancet (BD Microtainer, BD Benelux) by a trained veterinarian. The first capillary drop of blood was discarded. After collection, all liquid samples were stored at 4 °C, while VAMS samples were stored at ambient temperature in zip-lock bags containing desiccant, before transport to the Laboratory of Toxicology within 1 week (covered by stability data). There, whole blood and plasma were aliquoted per 50 µL for analysis. VAMS samples were dried for a minimum of 2 h. All samples were stored at -80 °C until analysis.

Iohexol and creatinine were determined in all samples (plasma, whole blood, vVAMS and cVAMS) using the developed and validated LC-MS/MS methods (as described in 2.1). The hct of the EDTA whole blood samples collected at t_0_ was determined using a Pro-Analytical C1015 centrifuge (Centurion Scientific, Chichester, United Kingdom).

#### Study outcomes

Results obtained for the quantification of iohexol and creatinine were compared to assess the (extent of) agreement between the different matrices via the generation of Bland-Altman plots and Passing-Bablok regression analysis using Medcalc software, version 20.021 (Ostend, Belgium). Four comparisons were made: (1) blood *versus* plasma; (2) vVAMS samples *versus* blood; (3) cVAMS *versus* vVAMS samples; and (4) cVAMS samples *versus* plasma. For creatinine, time-dependent differences between blood and plasma concentrations were evaluated via the generation of boxplots per sampling timepoint using Graphpad Prism 9 (version 9.0.2; Graphpad Software, San Diego, CA, USA). For comparison (2) and (4), an acceptance criterion of < 20% difference for 2/3 of the samples was taken, as recommended by Capiau et al. in the guideline for the development and validation of dried blood-based methods and by the EMA for incurred sample reanalysis [25, 26]. For the other direct comparisons, no acceptance criteria could be applied, as there may be an intrinsic difference between blood and plasma concentrations on the one hand, and between venous and capillary concentrations on the other hand.

As iohexol and creatinine reference concentrations are typically plasma-based, this implies that for clinical interpretation the results obtained from VAMS samples (dried blood) should ideally be convertible in a reliable manner to plasma concentrations. In this clinical validation study, we therefore assessed the correlation between concentrations in VAMS samples (blood) and corresponding plasma samples, using authentic patient samples and evaluated the need for a conversion factor. Different approaches can be used for conversion of a dried blood result to a plasma result, including hct-based conversion or conversion based on experimentally derived factors such as the Passing-Bablok regression fit, mean concentration ratio-based conversion and blood to plasma ratio-based conversion (Boffel et al. manuscript submitted). Since it is known that iohexol is only present in the plasma fraction of blood, we investigated the applicability of hct-based conversion using the hct determined at t_0_ for each individual cat. For creatinine, the applicability of conversion based on the Blood/Plasma (B/P) ratios derived from the clinical validation study was evaluated in a time-dependent manner. The derived conversion formulas were applied to an independent dataset to confirm their validity (see 2.3).

### Application

Finally, the developed methodologies were applied to samples derived from 40 cats who were recruited in the application study. The same inclusion criteria were applied as in the clinical validation study. From these 40 cats, only plasma and cVAMS samples were collected and analyzed according to the procedure described in 2.2.2. To validate the performance of the conversion formulas derived from the results of the clinical validation study (see 3.2.3), these formulas were applied to the independent cVAMS application data. For iohexol, the following conversion formula was used: $\:x\:\left(plasma\right)=\frac{y\:\left(cVAMS\right)}{\left(1-hct\right)\:\text{*}\:0.88}$, with hct representing the hct from the individual cat obtained from the whole blood sample collected at t_0_. For creatinine, the final conversion formula was the following: $\:x\:\left(plasma\:tx\right)=\frac{y\:\left(cVAMS\:tx\right)}{\left(\frac{\text{B}}{\text{P}}tx\right)\:\text{*}\:0.92}$, with $\:\frac{\text{B}}{\text{P}}tx$ representing the mean Blood/Plasma ratio determined in the clinical validation study for each time point of sample collection (t_0_, t_5_, t_30_, t_60_, t_120_, t_180_, t_360_ and t_600_). The mean B/P ratios for each time point can be found in Supplementary Table 3.

The plasma concentrations derived from cVAMS samples were compared to the corresponding plasma concentrations via the generation of Bland-Altman plots and Passing-Bablok analysis using Medcalc software, version 20.021. Also here, an acceptance criterion of < 20% difference for 2/3 of the samples was taken [25, 26].

## Results

### Analytical method development and validation

For the sample preparation for VAMS samples, the same precipitation solvent as for blood and plasma (MeOH) was tested initially. However, this did not yield a full precipitation of all proteins in all samples. More specifically -and surprisingly- human blood samples behaved differently compared to the samples prepared from cat blood. Therefore, different precipitation solvent compositions were evaluated, with 75/25 ACN/MeOH yielding a reproducible protein precipitation in both human and cat blood samples. Additionally, for an optimized precipitation, the counterweight used in the centrifuge had to be exactly identical.

**Fig. 2:**
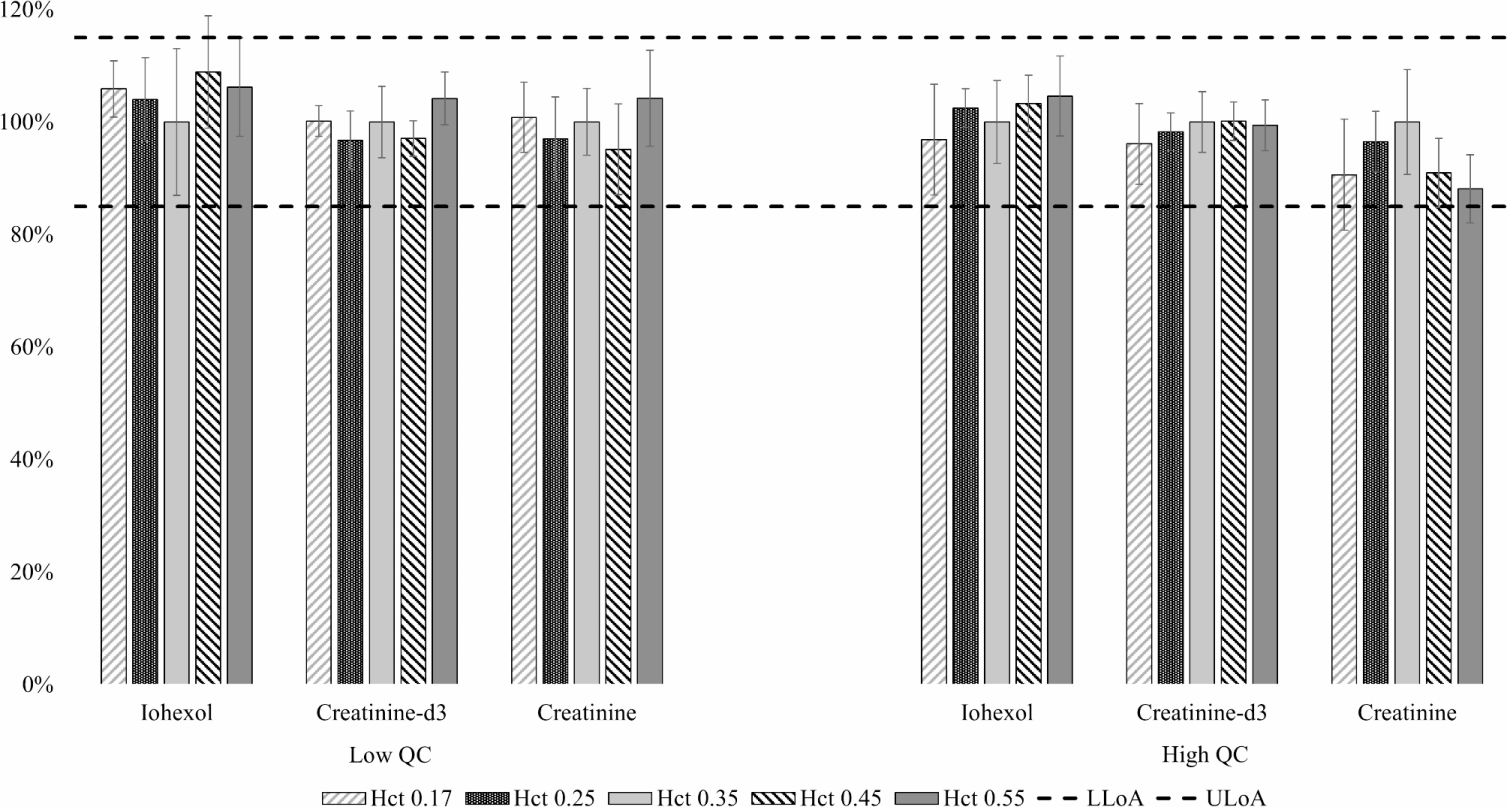
Effect of the hematocrit (hct) on the recovery (RE) from VAMS samples. The RE was determined from VAMS samples (*n* = 6) with hct values ranging from 0.17 to 0.55 L/L, and normalised to the RE at reference hct (0.35 L/L). The upper and lower limits of acceptance of ± 15% are represented by the dashed lines (LLoA; ULoA). The left and right part of the graph show the results (mean ± CV%) for the Low and High QC level, respectively. VAMS *volumetric absorptive microsampling*, CV *coefficient of variation*, QC *quality control*

To evaluate the robustness of the extraction of analytes from the VAMS samples, the automated extraction procedure was tested for a relevant effect of storage (‘ageing’) and the hct on the RE, as these factors may pose a challenge related to extractability [31]. Both the volume of the extraction solvent and the extraction time were evaluated. The final sample preparation procedure for VAMS samples is described in the Methods Sect. (2.1.4). To assess the effect of the hct, the RE was evaluated at two concentration levels, QCL and QCH, for five hct values across a broad range (from 0.17 to 0.55 L/L), with each condition being measured in sixfold. Figure 2 shows the data for the IS-compensated RE, relative to the RE of a reference hct of 0.35 L/L (set at 100%). No hct dependence was apparent and the RE was within the pre-set acceptance criterion at all hct levels, for both analytes.

The need for a correction factor to compensate for the use of creatinine-d3 calibrators was evaluated by the analysis of 29 plasma samples using creatinine-d3 calibration as well as using the reference measurement procedure for quantification of creatinine. The presence of systematic and proportional differences was evaluated via Passing-Bablok regression analysis (Supplementary Fig. 1). Both systematic and proportional differences were confirmed, as the 95% CI on the intercept and slope did not include 0 and 1, respectively (95% CI on intercept and slope, -0.91 to -0.32 and 0.75 to 0.80). Therefore, the obtained results were recalibrated to the reference measurement procedure targets in order to obtain a correct result using creatinine-d3 calibrators. The correction formula applied to correct the creatinine results is the equation of the Passing-Bablok regression analysis: y = -0.597 + 0.776 x. This correction formula was also applied to all the creatinine results in the clinical validation and application study.

All calibration models were linear with 1/x^2^ weighting. Back-calculation of the calibrators fulfilled the acceptance criterion, justifying the use of the chosen model. In Table 1, the accuracy and precision data for VAMS samples are shown for each QC level, including also QCL and QCH prepared in cat blood (indicated in bold). The bias was within 6% of the nominal concentration and both analytes could be determined with a repeatability and total imprecision below 9.6% (CV), meeting the pre-set acceptance criteria of 15% (20% at LLOQ). For blood and plasma, the accuracy and precision data are shown in Supplementary Table 4. Also here, all pre-set acceptance criteria were met, apart from an overall limited exceedance of the acceptance criterion for the total imprecision for creatinine-d3 at LLOQ in plasma and at QCL in blood (24.4% and 21.0% CV, respectively) and for iohexol at QCL in blood (19.2% CV).

Carry-over was within the pre-set limits of < 20% of the LLOQ area and < 5% of the IS area. For selectivity, the pre-dosing samples from the clinical validation study were assessed, with no unacceptable interferences observed. The selectivity for iohexol was within the pre-set limits of < 20% of the LLOQ area and < 5% of the IS area. For creatinine, the ion ratios in all matrices were within the tolerated window (± 20%) of the ion ratio in neat standard solution (ratio 25%).

**Table 1:** Accuracy (bias), repeatability (CV) and total imprecision (CV) data at the different QC levels for iohexol and creatinine-d3 in VAMS samples (*n* = 2 × 3) calculated via One-way ANOVA

		Nominal value (µg/mL)	Accuracy (bias)	Repeatability (CV)	Total imprecision (CV)
Iohexol	LLOQ	2	1.4%	8.0%	8.0%
	QCL	5	-2.9%	9.4%	9.4%
	QCM	50	-5.4%	0.9%	7.8%
	QCH	300	-0.5%	4.2%	7.2%
	**QCL cat**	**5**	**1.0%**	**5.5%**	**7.5%**
	**QCH cat**	**300**	**2.5%**	**5.3%**	**7.2%**
Creatinine-d3	LLOQ	2	-2.2%	8.3%	8.3%
	QCL	5	-3.3%	9.6%	9.6%
	QCM	50	-2.6%	2.5%	3.0%
	QCH	300	0.9%	2.8%	6.6%
	**QCL cat**	**5**	**1.1%**	**5.5%**	**8.0%**
	**QCH cat**	**300**	**2.8%**	**4.3%**	**6.2%**

QCL and QCH prepared in cat blood are indicated in bold. CV *coefficient of variation*, QC *quality control*, VAMS *volumetric absorptive microsampling*

The results of the ME and RE obtained in the different matrices are displayed in Supplementary Table 5. For iohexol, no noteworthy effect of the matrix on the ionization efficiency was observed, with absolute ME between 83% and 110%. However, for creatinine-d3 a substantial suppression of ionization (> 70% suppression in plasma) was observed. However, the IS-corrected ME all lay between 81% and 107%, indicating a good compensation of the IS for differences in ionization. Importantly, as creatinine(-d3) eluted early, incorporating a divert-to-waste step was essential to mitigate ME. The relative ME (CV) was below 6% in all cases, meeting the pre-set acceptance criterion of 15%. For the VAMS samples, including those at low and high hct, the relative ME lay below 5%, amply meeting the pre-set acceptance criterion. The absolute RE was relatively high in all cases (78 − 115%) and was reproducible, demonstrated by relative RE (CV) values below 8% and in this way also meeting the pre-set acceptance criterion of 15%.

The stability was investigated at the storage conditions relevant for the actual study samples, as displayed in Supplementary Table 6 for blood and plasma and in Supplementary Table 7 for VAMS samples. All analytes were stable in blood and plasma after storage for 1 week at 4 °C. Also, all analytes showed to be stable in VAMS devices stored for 1 week at RT. For iohexol and creatinine-d3 in VAMS devices, stability of the analytes for up to 2 days at 60 °C was demonstrated. As expected, a positive bias was found for creatinine after storage at elevated temperature, owing to conversion of creatine to creatinine following ring closure and the loss of a molecule of water of creatine to yield creatinine, as also found earlier [32, 33]. Remarkably, this positive bias was not observed for creatinine in the cat samples stored at elevated temperature.

The long-term stability at -80 °C was assessed longitudinally during the study sample analysis days. For plasma, stability was demonstrated for 8 months (time between t_0_ and t_5_), in blood for 3 months (time between t_0_ and t_2_) and in VAMS samples for 6 months (time between t_0_ and t_3_), as the % deviation from t_0_ was within 15% (except for a slight exceedance in VAMS at t_3_ at low level) (Supplementary Table 8).

The stability was also assessed for processed extracts, kept for 24–48 h in the autosampler at 10 °C. All samples were within 15% of the concentration found at t_0_ (except for creatinine at QCH in plasma (126% (24 h) and 128% (48 h)), overall justifying the reinjection of an analysis batch up to 48 h after the original analysis, when stored in the autosampler at 10 °C. No consistent or substantial degradation compared to t_0_ was found when freezing extracts at -20 °C for 14 days after the first analysis, subsequent thawing and reanalysis (except for 3 deviating values for creatinine: Cat QC3 in plasma, QCL in blood and QCH in a VAMS sample). A summary of the extract stability data can be found in Supplementary Table 9.

### Clinical method validation

Twenty-three cats were included in the clinical validation study. Their characteristics are described in Supplementary Table 10.

#### Iohexol

An overview of the results for both analytes per comparison can be found in Table 2.

**Fig. 3:**
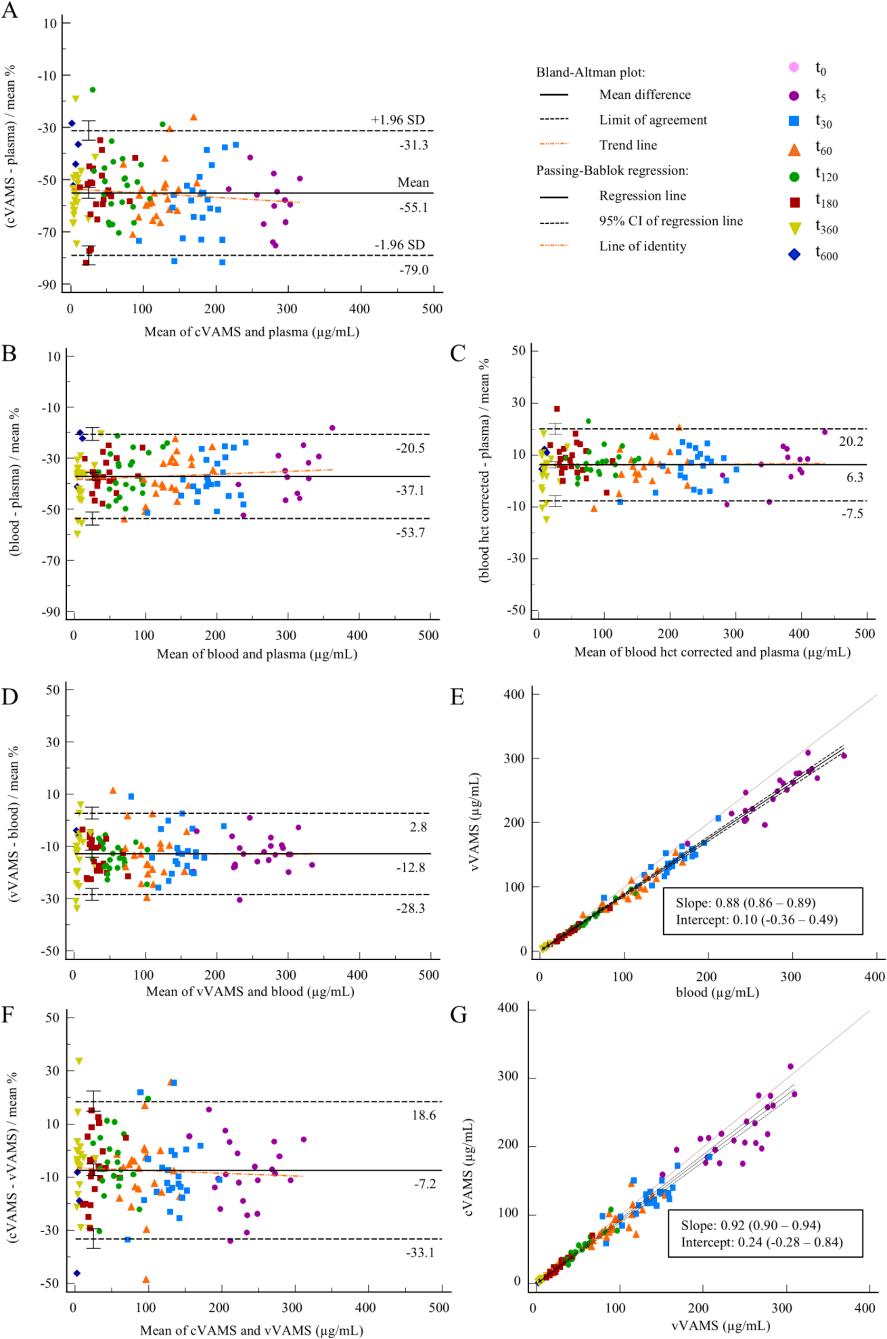
Bland-Altman plots for iohexol for the comparison of **A** cVAMS sample and plasma concentrations; **B** blood and plasma concentrations; **C** hematocrit (hct)-corrected blood and plasma concentrations; **D** vVAMS sample and blood concentrations; and **F** cVAMS and vVAMS sample concentrations. Passing-Bablok regression analysis for iohexol of **E** vVAMS sample concentrations plotted against blood concentrations; and **G** cVAMS sample concentrations plotted against vVAMS sample concentrations. VAMS *volumetric absorptive microsampling*, vVAMS *venous VAMS*, cVAMS *capillary VAMS*, CI *confidence interval*

For iohexol, a large mean bias of -55.1% (95% CI -57.3 to -53.0) was found when comparing the results for cVAMS and plasma samples (Fig. 3, Panel A). The limits of agreement (LoAs) lay at -79.0% and − 31.3% (Table 2). The differences between both matrices can be related to the different comparisons made in Fig. 3, Panels B-G.

When comparing blood and plasma results, a mean bias of -37.1% was found (95% CI -38.6 to -35.6) (Fig. 3, Panel B). The LoAs lay at -53.7% and − 20.5% (Table 2). Because it is known that iohexol shows negligible erythrocyte partitioning, it is typically quantified in plasma and hence the presence of erythrocytes in blood yields a dilution effect. This dilution effect can be estimated by the blood hct. Therefore, we corrected the iohexol blood results for the hct by using the following correction formula: $\:x\:\left(plasma\right)=\frac{y\:\left(blood\right)}{\left(1-hct\right)\:}$, using the hct of the blood sample drawn at t_0_ for each individual cat. When comparing these corrected blood concentrations to the corresponding plasma concentrations, a mean bias of 6.3% remained (95% CI 5.1 to 7.6) (Fig. 3, Panel C). The LoAs lay at -7.5% and 20.2% (Table 2).

**Table 2:** Overview of the results per comparison of the different matrices for iohexol and creatinine

		Iohexol	Creatinine
cVAMS vs. plasma	Mean difference (%) [95% CI] LoAs (%) [95% CI]	-55.1 [-57.3 to -53.0] -79.0 [-82.6 to -75.4] -31.3 [-34.9 to -27.6]	-21.2 [-23.2 to -19.2] -48.0 [-51.4 to -44.6] 5.5 [2.1 to 8.9]
Blood vs. plasma	Mean difference (%) [95% CI] LoAs (%) [95% CI]	-37.1 [-38.6 to -35.6] -53.7 [-56.2 to -51.1] -20.5 [-23.1 to -18.0]	-9.3 [-10.7 to -8.0] -27.2 [-29.4 to -24.9] 8.5 [6.2 to 10.8]
Blood hct-corrected vs. plasma	Mean difference (%) [95% CI] LoAs (%) [95% CI]	6.3 [5.1 to 7.6] -7.5 [-9.7 to -5.4] 20.2 [18.1 to 22.4]	
vVAMS vs. blood	Mean difference (%) [95% CI] LoAs (%) [95% CI] Number of samples within ± 20% difference [%]	-12.8 [-14.1 to -11.4] -28.3 [-30.6 to -26.0] 2.8 [0.5 to 5.1] 111/135 [82%]	-7.2 [-8.4 to -6.0] -23.3 [-25.4 to -21.3] 8.9 [6.8 to 10.9] 168/181 [93%]
cVAMS vs. vVAMS	Mean difference (%) [95% CI] LoAs (%) [95% CI]	-7.2 [-9.5 to -5.0] -33.1 [-36.9 to -29.3] 18.6 [14.8 to 22.4]	-4.9 [-6.7 to -3.1] -28.6 [-31.6 to -25.6] 18.8 [15.8 to 21.9]

CI *confidence interval*, cVAMS *capillary VAMS sample*, LoAs *limits of agreement*, VAMS *volumetric absorptive microsampling*, vVAMS *venous VAMS sample*

When comparing the concentrations from vVAMS and blood samples (Fig. 3, Panel D), a mean bias of -12.8% was observed (95% CI -14.1 to -11.4) and the LoAs lay at -28.3% and 2.8% (Table 2). Whereas the acceptance criterion of < 20% mean difference for two-thirds of the samples, as recommended by Capiau et al. [26], was formally fulfilled for iohexol (with 111/135 (82%) of the differences being < 20%), Passing-Bablok regression showed significant proportional differences (95% CI on slope 0.86 to 0.89), whereas no systematic differences were apparent (95% CI on intercept − 0.36 to 0.49) (Fig. 3, Panel E).

Upon comparing the concentrations of vVAMS and cVAMS samples **(**Fig. 3, **Panel F)**, a mean difference of -7.2% was observed (95% CI -9.5 to -5.0), with LoAs laying at -33.1% and 18.6% **(**Table 2**)**. Passing-Bablok regression analysis revealed a slight, but significant proportional error (95% CI on slope 0.90 to 0.94), while no systematic differences could be observed (95% CI on intercept − 0.28 to 0.84) **(**Fig. 3, **Panel G)**.

Overall, the differences observed in these different comparisons indicated the need to apply conversion formulas to reliably derive plasma concentrations from (dried) whole blood (whether venous or capillary) concentrations.

#### Creatinine

**Fig. 4:**
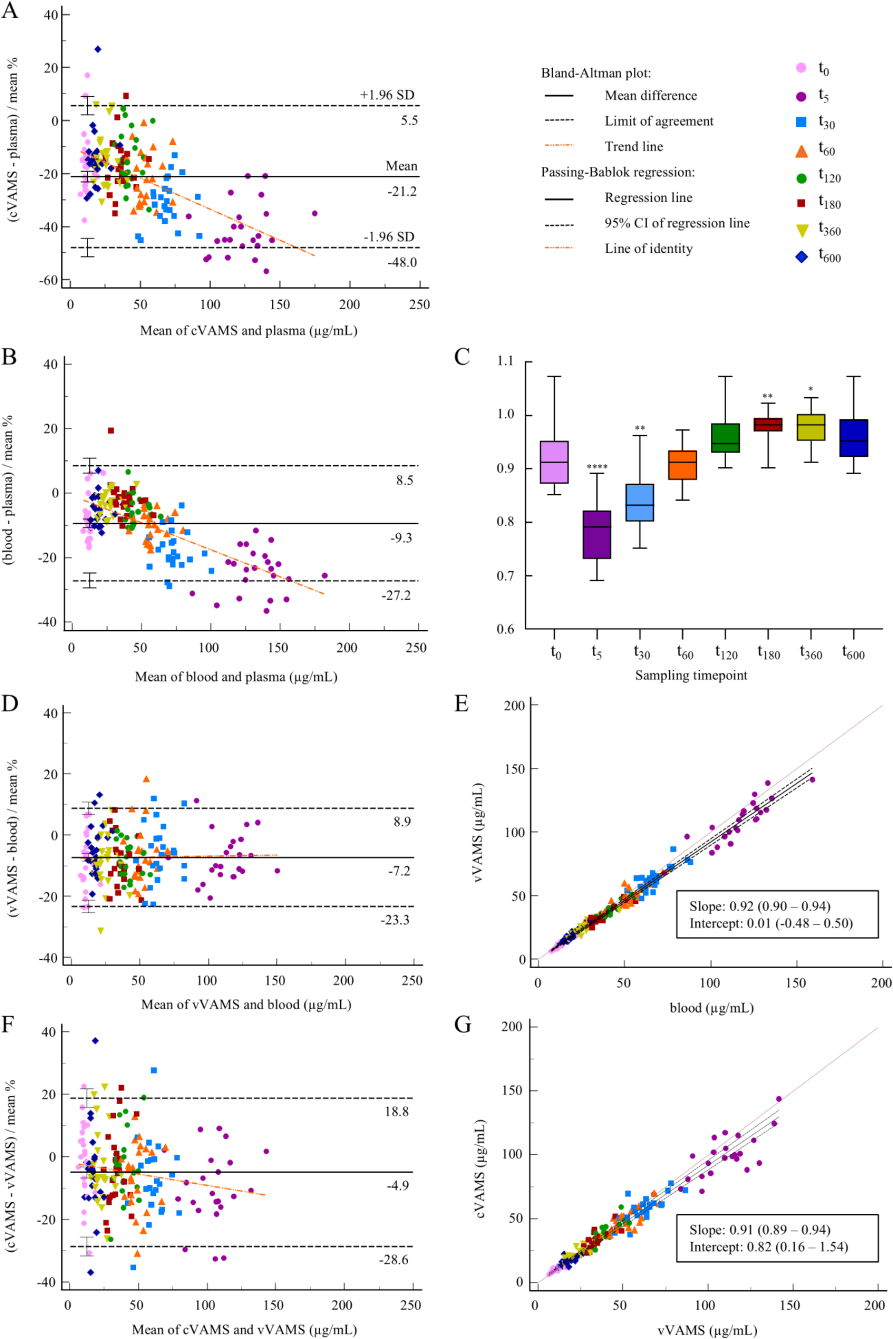
Bland-Altman plots for creatinine for the comparison of **A** cVAMS sample and plasma concentrations; **B** blood and plasma concentrations; **D** vVAMS sample and blood concentrations; and **F** cVAMS and vVAMS sample concentrations. Boxplots of **C** creatinine B/P ratios per sampling time point. The boxes indicate the 25th and 75th percentile and median, and the flags show the minimum and maximum values. Single, double and quadruple asterisks (*, ** and ****) indicate significant differences from the median of t_0_ (Dunn’s multiple comparison test, α = 0.05). Passing-Bablok regression analysis for creatinine of **E** vVAMS sample concentrations plotted against blood concentrations; and **G** cVAMS sample concentrations plotted against vVAMS sample concentrations. VAMS *volumetric absorptive microsampling*, vVAMS *venous VAMS*, cVAMS *capillary VAMS*, CI *confidence interval*

Comparison of the creatinine results obtained for cVAMS and plasma samples revealed a mean bias of -21.2% (95% CI -23.2 to -19.2) (Fig. 4, Panel A). A significant, negative slope of -0.23 (95% CI -0.28 to -0.19) was found. The LoAs lay at -48.0% and 5.5% (Table 2). The differences between the results of cVAMS and plasma samples can be related to the different comparisons displayed in Fig. 4, Panels B-G.

When comparing blood and plasma results, a mean bias of -9.3% (95% CI -10.7 to -8.0) and a significant, negative slope of -0.17 (95% CI -0.19 to -0.14) were found (Fig. 4, Panel B). The LoAs lay at -27.2% and 8.5% (Table 2). The median B/P ratios per sampling time point are displayed in Fig. 4, Panel C. The median B/P ratios at t_5_, t_30_, t_180_ and t_360_ were significantly different from the ratio at t_0_ (before administration of creatinine), indicating a time-dependent change in B/P ratio after exogenous administration of creatinine.

Upon comparing the results obtained for vVAMS samples and blood, a mean bias of -7.2% (95% CI -8.4 to -6.0) was found (Fig. 4, Panel D), with LoAs laying at -23.3% and 8.9% (Table 2). With 168/181 (93%) of the differences being < 20%, the acceptance criterion of < 20% mean difference for two-thirds of the samples, was fulfilled for creatinine. However, Passing-Bablok regression showed slight, but significant proportional differences (95% CI on slope 0.90 to 0.94), whereas no systematic differences were apparent (95% CI on intercept − 0.48 to 0.50) (Fig. 4, Panel E).

Comparison of cVAMS and vVAMS sample results showed a mean bias of -4.9% (95% CI -6.7 to -3.1) (Fig. 4, Panel F). The LoA lay at -28.6% and 18.8% (Table 2). A slight, but significant slope of -0.07 (95%CI -0.13 to -0.01) was apparent. Passing-Bablok regression analysis resulted in slight, but significant proportional and systematic differences (95% CI on slope 0.89 to 0.94, 95% CI on intercept 0.16 to 1.54) (Fig. 4, Panel G).

Overall, also for creatinine the differences observed in these different comparisons indicated the need to apply conversion formulas to reliably derive plasma concentrations from (dried) whole blood (whether venous or capillary) concentrations.

#### Conversion formulas

To allow clinical interpretation of the cVAMS results for iohexol and creatinine, these should be converted to a plasma concentration, as reference concentrations are typically determined in plasma. Based on the results of the clinical validation study, we established conversion formulas to convert the results of cVAMS samples to ‘calculated plasma concentrations’.

For iohexol, we evaluated hct-based conversion using the hct determined at t_0_ for each individual cat. Additionally, because we found a significant proportional difference between vVAMS samples and blood results (Fig. 3, Panel E), we incorporated the slope of 0.88 as a constant factor in the conversion formula to compensate for this. The final conversion formula was the following: $\:x\:\left(plasma\right)=\frac{y\:\left(cVAMS\right)}{\left(1-hct\right)\:\text{*}\:0.88}$, with the hct corresponding to the whole blood hct from the individual cat at t_0_. The validity of this conversion formula was to be confirmed by applying it to the independent application dataset (see 2.3).

Based on Bland-Altman analysis for creatinine, 9.3% lower blood results compared to plasma were obtained (Fig. 4, Panel B). The lower result in blood can be explained by a dilution factor in blood, compared to plasma. In order to obtain plasma concentrations from cVAMS concentrations, we evaluated a conversion based on the B/P ratio. While doing so, we noticed a time-dependent change in B/P ratio after administration (Fig. 4, Panel C). Therefore, the B/P ratios were determined for each individual sampling time point based on the clinical validation data. The mean B/P ratios and CV (%) are reported in Supplementary Table 3. Because we also found a significant proportional bias between the creatinine results obtained for vVAMS samples and blood (Fig. 4, panel E), the slope of 0.92 was incorporated as a constant factor in the conversion formula to take this into account. As for creatinine, the difference between the cVAMS and plasma results was not dependent on the hct, no hct-dependent conversion was required. The final conversion formula derived from the clinical validation data that was to be validated via the independent application dataset (see 2.3) was the following: $\:x\:\left(plasma\:tx\right)=\frac{y\:\left(cVAMS\:tx\right)}{\left(\frac{\text{B}}{\text{P}}tx\right)\:\text{*}\:0.92}$, with $\:\frac{\text{B}}{\text{P}}tx$ representing the mean B/P ratio for each time point of sample collection (t_0_, t_5_, t_30_, t_60_, t_120_, t_180_, t_360_ and t_600_).

### Application

Forty cats were included in the application study. Their characteristics are described in Supplementary Table 10.

To validate the conversion formulas for iohexol and creatinine described in 3.2.3, these were applied on cVAMS samples collected from 40 different cats, recruited in the framework of a study aiming at evaluating the applicability of ear-prick sampling for GFR determination. These converted cVAMS results (expressed as ‘calculated plasma concentrations’) were compared to those obtained for the corresponding venous plasma samples, in which the concentration had been directly measured.

**Fig. 5:**
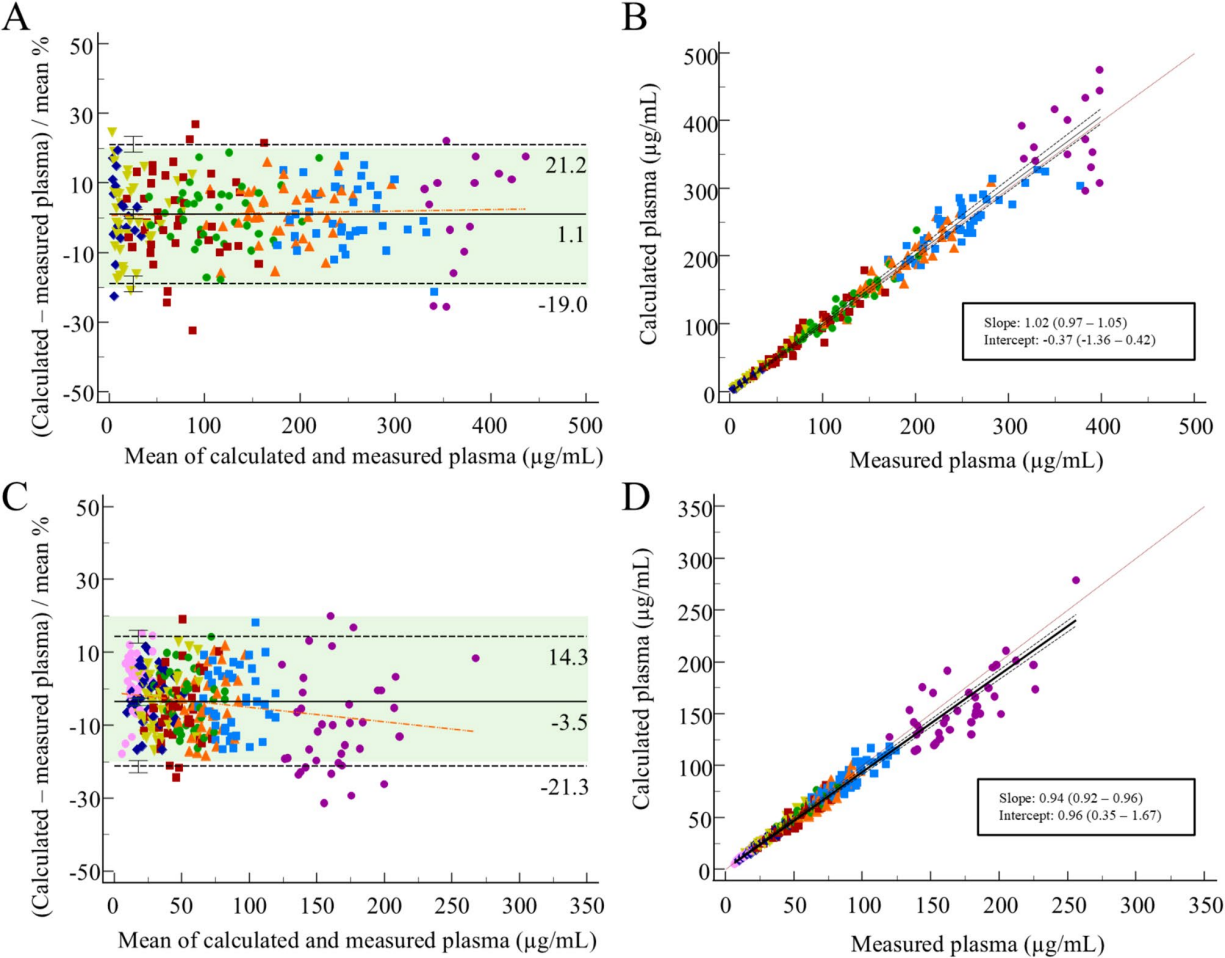
Bland-Altman plots for the comparison of **A** calculated (cVAMS-based) and measured plasma iohexol concentrations; and **C** calculated (cVAMS-based) and measured plasma creatinine concentrations. Passing-Bablok regression analysis of **B** calculated (cVAMS-based) plasma concentrations plotted against measured plasma concentrations for iohexol; and **D** calculated (cVAMS-based) plasma concentrations plotted against measured plasma concentrations for creatinine. VAMS *volumetric absorptive microsampling*, cVAMS *capillary VAMS*

For iohexol, the comparison of calculated plasma concentrations and the corresponding measured plasma concentrations revealed a minimal and non-significant mean bias of 1.1% (95% CI -0.2 to 2.4), with no concentration-dependent effects (Fig. 5, Panel A). The LoAs lay at -19.0% and 21.2% (Table 3). The acceptance criterion of < 20% mean difference for two-thirds of the samples was easily met with 220/233 (94%) of the differences being < 20%. Passing-Bablok regression analysis revealed neither systematic nor proportional differences (95% CI on intercept − 1.36 to 0.42, 95% CI on slope 0.97 to 1.05) (Fig. 5, Panel B).

For creatinine, Bland-Altman comparison between calculated plasma concentrations and the corresponding measured plasma concentrations revealed a small yet significant mean bias of -3.5% (95% CI -4.5 to -2.5), with a slight concentration dependence, as indicated by a small but significant slope of the trend line between the differences, of -0.04 (95% CI -0.06 to -0.02) (Fig. 5, Panel C). The LoAs lay at -21.3% and 14.3% (Table 3). For creatinine, 307/320 (96%) of the differences were < 20%, amply meeting the acceptance criterion as recommended by Capiau et al. Both (slight) systematic and proportional differences were obtained via Passing-Bablok regression analysis (95% CI on intercept 0.35 to 1.67, 95% CI on slope 0.92 to 0.96) (Fig. 5, Panel D).

**Table 3:** Overview of the results for the comparison of calculated plasma concentrations vs. measured plasma concentrations for iohexol and creatinine

		Iohexol	Creatinine
Calculated plasma concentrations* vs. measured plasma concentrations	Mean difference (%) [95% CI] LoAs (%) [95% CI] Number of samples within ± 20% difference [%]	1.1 [-0.2 to 2.4] -19.0 [-21.2 to -16.7] 21.2 [18.9 to 23.4] 220/233 [94%]	-3.5 [-4.5 to -2.5] -21.3 [-23.1 to -19.6] 14.3 [12.6 to 16.1] 307/320 [96%]

*: calculated from capillary volumetric absorptive microsampling (cVAMS) samples, CI *confidence interval*, LoA *limits of agreement*

## Discussion

In this study, we developed, optimized and analytically validated novel LC-MS/MS methods for the simultaneous quantification of iohexol and creatinine in blood, plasma and VAMS samples. Additionally, the goal was to assess whether VAMS-based ear-prick sampling could potentially be used for future GFR determination based on both iohexol and creatinine clearance, following conversion of cVAMS results to plasma results. This is relevant to further minimize the invasiveness and potentially also stress for the cats and to increase the practical feasibility of the otherwise cumbersome GFR sampling procedure.

First, LC-MS/MS assays for the different matrices (venous plasma and blood, vVAMS and cVAMS samples) were successfully developed, optimized and analytically validated. Special attention was paid to the sustainability of the procedure by minimizing the use of cat blood – both for the validation and the eventual application.

The amount of cat samples in the analytical validation was kept as low as possible by preparing calibrators for the quantification of the study samples in human-based matrices instead of in cat-based matrices. This is highly relevant, as larger quantities of human blood are much more easy to obtain than larger quantities of cat blood. However, this involved a thorough evaluation and optimization. E.g., we encountered an unanticipated difference in protein precipitation in human blood compared to cat blood, which required optimization of the precipitation solvent (decreasing the polarity by increasing the % acetonitrile compared to the % methanol). One possible explanation is that, while the general types of proteins in human and cat blood are similar, the concentrations and specific forms of these proteins may differ due to species-specific physiology [34]. To confirm and validate the matrix compatibility, accuracy and precision of the methods were also assessed in cat blood samples. An excellent accuracy was obtained for VAMS samples prepared from cat blood, with a maximum bias of 2.8%. The method also proved to be precise in cat blood VAMS samples, with intra-day precision and total imprecision below 5.5% and 8%, respectively. The pre-set acceptance criteria for accuracy and precision were easily met, in this way also indirectly demonstrating acceptable ME and RE in samples prepared from cat blood. As such, the validity of calibration in human blood (which is more readily available) for quantification of cat blood samples was confirmed.

A second aspect of the sustainable nature of the procedure lies in the use of VAMS samples, only requiring 10 µL of cat blood via a minimally invasive ear-prick. An important disadvantage of a dried blood matrix is the requirement of a more elaborate sample preparation procedure. To overcome this hurdle, we developed an automated extraction procedure using the Waters Andrew + pipetting robot. Importantly, as we were aware of a potential effect of the hct on the RE of the analytes, especially when using an automated extraction procedure [35], we evaluated the robustness of the method for this hct effect more into depth. The hct independence was demonstrated by relative RE values within the pre-set acceptance criterion across a broad hct range from 0.17 to 0.55 L/L (covering the hct range of the study population). Similarly, using a manual extraction procedure, Dhondt et al. showed that iohexol can be quantified reliably in VAMS samples with hct values between 0.20 and 0.60 L/L [21]. The robust, automated extraction procedure reported here allows high-throughput analysis of VAMS samples.

Following analytical method validation, the method was clinically validated to evaluate the interchangeability of the ‘calculated plasma concentrations’, derived from the VAMS-based ear-prick sampling, and the concentrations in (venous) plasma, i.e. the conventional sample used for GFR measurements. This clinical validation encompassed the comparison and statistical evaluation of paired VAMS, venous blood and plasma samples [26]. For the comparison of the concentrations of cVAMS and plasma samples, for both iohexol and creatinine a large negative bias of -55.1% and − 21.2% was found, respectively. However, when comparing these two matrices, multiple variables are evaluated and should be taken into account: (1) intrinsic differences between blood and plasma concentrations, (2) the effect of the use of VAMS as a sampling technique and (3) the effect of capillary *versus* venous sampling. As a result, the differences observed between cVAMS and plasma results reflect a combination of all these factors and can be further analyzed through the remaining comparisons made in the clinical validation study.

First, for iohexol, when comparing blood and plasma concentrations, a large negative bias of -37.1% was found. This was expected and in alignment with previous reports describing that iohexol shows negligible erythrocyte partitioning and is hence mainly present in the plasma fraction of blood [36, 37]. When comparing blood to plasma results, the presence of erythrocytes yields a dilution effect which can be approximated by the blood hct. The mean hct of the study population was 35.5% and explains the 37.1% higher concentrations found in plasma compared to blood. When correcting the blood result according to the cat’s individual hct (determined at t_0_) the bias becomes 6.3% (95% CI 5.1 to 7.6). This slight ‘overcorrection’ might be explained by an overestimation of the hct at an individual level for the later time points, when using only the hct determined at t_0_ for correction of all samples (at all time points) for an individual cat. Because a substantial total amount of blood is drawn from the cat during the procedure (one of the reasons to evaluate a microsampling-assisted procedure), this might have a slight influence on the hct at the end of the procedure.

For creatinine, the blood results were on average 9.3% lower than those in plasma. At baseline (t_0_), blood concentrations were 7.6% lower than in plasma. This observation is in line with literature [32, 33], with the lower blood results being attributable to a dilution factor in blood, compared to plasma, although less pronounced than for iohexol. Additionally and importantly, a significant concentration-dependent trend was observed, indicating that the bias increases with the concentration. When plotting the B/P ratio for creatinine at each time point of sample collection, a significant decrease compared to the ratio at t_0_ (only endogenous creatinine) can be observed after administration of exogenous creatinine. This ratio gradually normalized again, until reaching an equilibrium at the ratio initially observed for endogenous creatinine. This concentration-dependent trend (negative bias in samples with higher creatinine concentration, collected at early sampling time points) indicates that it takes some time before the exogenously administered creatinine reaches an equilibrium in terms of blood-plasma distribution.

Comparison of the concentrations measured in vVAMS samples with those measured in whole blood should point out whether the VAMS approach *in se* has an influence on the determination of iohexol and creatinine. This appeared to be the case, as a negative bias of -12.8% and − 7.2% was observed for iohexol and creatinine, respectively, indicating that the results obtained for vVAMS samples are lower than the corresponding blood results. Also Passing-Bablok regression analysis revealed significant proportional differences for these comparisons (slope of 0.88 for iohexol and 0.92 for creatinine). A possible explanation for this might be that in real, authentic cat samples the extractability differs somewhat from the extractability of artificial, ‘spiked’ samples. An extraction procedure, evaluated and optimized using spiked samples, might still not be fully optimal for the extraction of authentic samples and further optimization might still be possible. Of note, the differences can still be considered acceptable since the acceptance criterion was still met for both iohexol and creatinine (82% and 93% of the differences within ± 20%, respectively). Importantly, the limited spread of the observed differences indicated that this ‘methodological bias’ was consistent, rendering it possible to correct for this difference in a next step.

The effect of capillary *versus* venous sampling was evaluated by comparing the results from VAMS samples prepared from venous blood and VAMS samples prepared from paired capillary blood obtained via ear-prick sampling. For both analytes, a negative bias was found (-7.2% for iohexol and − 4.9% for creatinine). Importantly, the span between the LoAs for the comparison of cVAMS *versus* vVAMS samples is wider than for the comparison of vVAMS samples *versus* liquid blood (51.7% vs. 31.1% for iohexol and 47.4% vs. 32.2% for creatinine, respectively). Also slight, but significant proportional differences were found via Passing-Bablok regression analysis (slope: 0.92 for iohexol and 0.91 for creatinine). These differences and wider spans can primarily be explained by the influence of the first time point samples after administration (t_5_), as there was inherently always a small delay between the venous blood collection and the ear-prick. Indeed, capillary-venous differences can be anticipated to be more pronounced by a slight delay during the early phase, in which there is a steep decline in the concentration-time curve. Another factor contributing to the wider span might be a somewhat larger imprecision for the analysis of cVAMS samples compared to vVAMS samples generated in the laboratory, as also found earlier by our own group [38–40]. For iohexol, the group of Zwart et al. also found a capillary-venous divergence for early timed samples, which they attributed to a possible incomplete capillary distribution of iohexol at these time points [19].

Considering all the factors contributing to the differences between cVAMS and plasma samples, and the fact that for clinical interpretation plasma-based results are required, it is clear that a conversion formula is needed to convert the results from cVAMS samples to plasma concentrations. For iohexol, we used individual hct-based conversion, as already applied by other groups as well [13, 14, 16, 36, 37]. For creatinine, we performed a conversion based on the B/P ratio, as evaluated earlier by our group [33]. Moreover, as a time-dependent trend was found for the B/P ratio, we used the B/P ratios determined at each individual sampling time point to perform a ‘time-dependent’ conversion. Additionally, for both analytes, a constant factor was included in the conversion formula to compensate for the ‘methodological bias’ we observed when comparing the results from vVAMS samples and liquid blood. The established conversion formulas were validated by applying them on an independent dataset containing cVAMS samples and plasma from 40 cats (application study). For both analytes, an excellent agreement was obtained between the calculated (cVAMS-based) and measured plasma concentrations, with a narrow span between the LoA’s. With 94% and 96% of the differences lying within 20% difference for iohexol and creatinine, respectively, the acceptance criterion was amply met. Consequently, our study demonstrates that iohexol and creatinine can be quantified accurately and precisely starting from a cVAMS sample and that, following conversion, the obtained results can be used interchangeably with venous plasma data (the reference). To further evaluate the clinical applicability of (capillary) VAMS sampling, the GFR results calculated from cVAMS samples should be compared to those calculated from the plasma samples (reference) and the impact on the classification (stages) of the patient’s kidney function should be assessed.

## Conclusion

We successfully developed, optimized and validated LC-MS/MS assays for the simultaneous quantification of iohexol and creatinine in blood, plasma and VAMS samples. A key challenge was enhancing the ethical and sustainable aspects of the study, which we addressed by reducing reliance on cat blood-based matrices and incorporating microsampling techniques. Comprehensive evaluation of bio-analytical and VAMS-specific validation parameters confirmed compliance with the pre-set validation acceptance criteria.

Through a clinical validation study, we established conversion formulas to reliably obtain cVAMS-derived plasma results, which closely matched directly measured plasma results for both iohexol and creatinine. In conclusion, we demonstrated that ear-prick sampling using VAMS provides a practical and suitable alternative to conventional venous sampling for measuring iohexol and creatinine for GFR estimation in cats.

This VAMS-based sampling protocol not only meets the practitioner’s need for a more convenient method but also enhances animal welfare and ethical standards. Furthermore, it facilitates GFR measurements, which -given the burden of conventional sampling to the cats-are not typically performed in routine practice, enabling early detection of kidney function decline and improving the prognosis for cats.

## Electronic supplementary material

Below is the link to the electronic supplementary material.

Supplementary Material 1

## Data Availability

Data is provided within the manuscript or supplementary information files.

## Abbreviations

3R: Reduce refine replace
CAN: Acetonitrile
ANOVA: Analysis of variance
B/P: Blood/Plasma
CI: Confidence interval
CKD: Chronic kidney disease
CV: Coefficient of variation
cVAMS: Capillary VAMS
DBS: Dried blood spots
EMA: European Medicines Agency
ESI: Electrospray ionization
FA: Formic acid
FDA: U.S Food and Drug Administration
GFR: Glomerular filtration rate
Hct: Hematocrit
IATDMCT: International Association of Therapeutic Drug Monitoring and Clinical Toxicology
IRIS: International Renal Interest Society
IS: Internal standard(s)
LC-(MS/)MS: liquid chromatography - (tandem) mass spectrometry
LLOQ: Lower limit of quantification
LoA(s): Limit(s) of agreement
ME: Matrix effect
MeOH: Methanol
MP: Mobile phase
MRM: Multiple reaction monitoring
QC(L/M/H): Quality control low / medium / high
RE: Recovery
RT: Room temperature
VAMS: Volumetric absorptive microsampling
vVAMS: Venous VAMS
