# Supplementary material for: Volumetric absorptive microsampling to measure iohexol and creatinine concentrations for estimation of glomerular filtration rate in cats: aligning animal welfare with practical feasibility
Source: BMC Vet Res. 2025 Apr 27;21:294. doi: 10.1186/s12917-025-04748-2 (PMC12034177; doi:10.1186/s12917-025-04748-2)
Supplement: Supplementary file 1 — Supplementary Material 1 [file 12917_2025_4748_MOESM1_ESM.docx]

**Additional File 1**


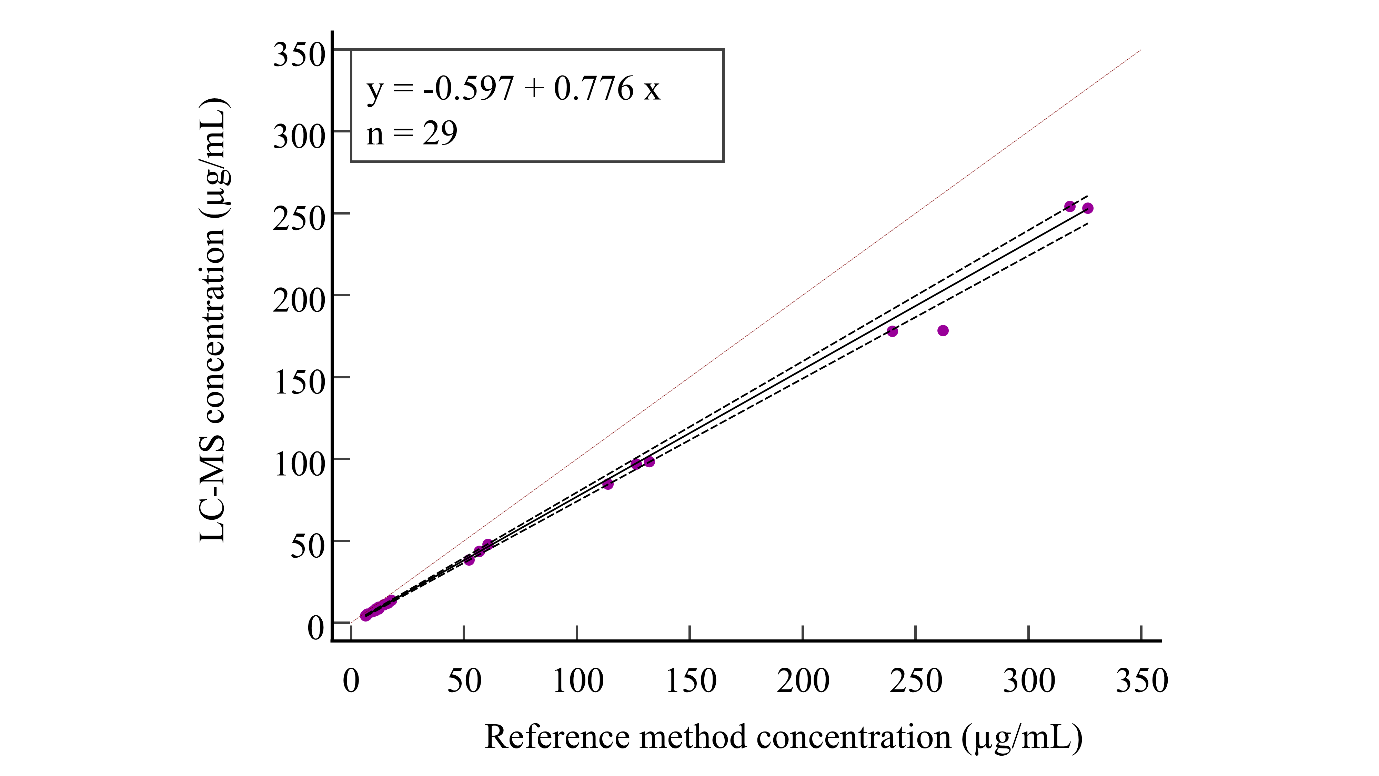


**Supplementary Figure 1** Passing-Bablok analysis to obtain a correction factor for creatinine, with a = 0.776 and b = -0.597. LC-MS/MS plasma concentrations plotted in function of reference method plasma concentrations. The red line represents the line of equality. The black line indicates the regression line (broken black lines 95% CI).

**Supplementary Table 1** MRM transitions and compound-specific mass spectrometric parameters for iohexol, creatinine and creatinine-d3 and the corresponding internal standards.

|  | Precursor ion (m/z) | Product ion (m/z) | Cone (V) | Collision (V) |
| --- | --- | --- | --- | --- |
| **Iohexol**  **Iohexol-d5**  **Creatinine**  **Creatinine-d3**  **Creatinine-13C3d3** | 821.98  821.98  826.98  114.07  114.07  117.07  117.07  120.10 | 804.05  375.06  809.05  44.02  86.10  47.02  89.10  49.14 | 64  64  64  68  68  68  68  68 | 18  38  18  12  8  12  8  12 |

**Supplementary Table 2** Criteria for inclusion of cats in the different groups.

| **Group 1: Healthy aged** |
| --- |
| - Older than 7 years of age - ‘Healthy’ according to the owner - No clinically relevant abnormalities based on physical examination, blood pressure measurement, extended blood-and urinalysis, FeLV/FIV test and abdominal ultrasound   **Group 2: Non-azotemic CKD**   - Serum creatinine and urea are below upper reference interval and one or more of the criteria described for IRIS Stage 1 CKD are present:   - positive trend serum creatinine within the reference interval (increase ≥ 30%)   - positive trend SDMA (symmetric dimethylarginine) within the reference interval (increase ≥ 50%)   - 2 x USG (urine specific gravity) < 1.035 (non-renal causes excluded)   - 2 x urine protein creatinine ratio (UPC) > 0.4 (non-renal causes excluded)   - 2 x SDMA = 15-17 mg/dL   - Significant renal abnormality on abdominal ultrasound |
| **Group 3: Azotemic CKD** |
| - Persistent renal azotemia and USG below 1.035 |

**Supplementary Table 3** Blood to plasma ratios determined at each timepoint of sample collection based on 23 paired samples.

|  | Blood/Plasma ratio | | CV (%) |
| --- | --- | --- | --- |
| **t_0_**  **t_5_**  **t_30_**  **t_60_**  **t_120_**  **t_180_**  **t_360_**  **t_600_** | | 0.92  0.79  0.84  0.91  0.95  0.99  0.97  0.95 | [6.9]  [7.0]  [6.3]  [4.2]  [4.3]  [5.8]  [3.4]  [4.6] |

**Supplementary Table 4** Accuracy (bias), repeatability (CV) and total imprecision (CV) data at the different QC levels for iohexol and creatinine-d3 in plasma and blood samples (n = 2 x 3) calculated via One-way ANOVA. QCL and QCH prepared in cat blood are indicated in bold.

|  |  |  |  | Nominal value (µg/mL) | Accuracy (bias) | Repeatability (CV) | Total imprecision (CV) |
| --- | --- | --- | --- | --- | --- | --- | --- |
| Plasma | Iohexol | LLOQ |  | 2 | -8.7% | 7.1% | 12.0% |
|  |  | QCL |  | 5 | -9.3% | 1.9% | 9.0% |
|  |  | QCM |  | 50 | -7.6% | 1.1% | 6.3% |
|  |  | QCH |  | 300 | -4.7% | 1.6% | 7.8% |
|  |  | **QCL cat** |  | **5** | **-6.7%** | **6.5%** | **9.9%** |
|  |  | **QCH cat** |  | **300** | **-2.2%** | **2.6%** | **8.1%** |
|  | Creatinine-d3 | LLOQ |  | 2 | -13.5% | 11.2% | 24.4% |
|  |  | QCL |  | 5 | -10.3% | 5.4% | 8.1% |
|  |  | QCM |  | 50 | -6.5% | 2.6% | 4.2% |
|  |  | QCH |  | 300 | 1.1% | 4.9% | 4.9% |
|  |  | **QCL cat** |  | **5** | **-6.7%** | **10.0%** | **10.0%** |
|  |  | **QCH cat** |  | **300** | **5.2%** | **3.9%** | **5.0%** |
| Blood | Iohexol | LLOQ |  | 2 | -10.3% | 3.8% | 8.5% |
|  |  | QCL |  | 5 | 6.4% | 3.5% | 19.2% |
|  |  | QCM |  | 50 | -7.9% | 2.2% | 9.5% |
|  |  | QCH |  | 300 | -8.0% | 3.4% | 3.4% |
|  |  | **QCL cat** |  | **5** | **-6.3%** | **4.3%** | **9.1%** |
|  |  | **QCH cat** |  | **300** | **-0.3%** | **3.1%** | **6.1%** |
|  | Creatinine-d3 | LLOQ |  | 2 | -8.7% | 4.8% | 10.4% |
|  |  | QCL |  | 5 | 8.7% | 4.6% | 21.0% |
|  |  | QCM |  | 50 | -9.5% | 1.8% | 6.6% |
|  |  | QCH |  | 300 | -6.1% | 3.2% | 3.2% |
|  |  | **QCL cat** |  | **5** | **-13.5%** | **5.7%** | **7.6%** |
|  |  | **QCH cat** |  | **300** | **-7.0%** | **4.8%** | **5.1%** |

**Supplementary Table 5** Matrix effects and recovery of iohexol and creatinine-d3 in plasma, blood and VAMS samples at Low and High QC level (QCL and QCH). Matrix effects and recovery for plasma and blood were determined on samples from 6 individual donors. Matrix effects and recovery for VAMS samples were determined on blood from 6 individual donors and 1 additional low and high hematocrit blood sample.

|  |  |  | **Matrix effects** | | |  | **Recovery** | |
| --- | --- | --- | --- | --- | --- | --- | --- | --- |
|  |  |  | Absolute (%) | IS-corrected (%) | Relative (CV; %) |  | Absolute (%) | Relative (CV; %) |
| **Plasma** | **Iohexol** | **QCL** | 109.5 | 98.2 | [4.2] |  | 81.9 | [4.3] |
|  |  | **QCH** | 105.9 | 85.4 | [2.0] |  | 86.5 | [4.6] |
|  |  |  |  |  |  |  |  |  |
|  | **Creatinine-d3** | **QCL** | 30.5 | 84.2 | [5.9] |  | 100.3 | [7.2] |
|  |  | **QCH** | 23.0 | 81.1 | [2.0] |  | 96.4 | [4.9] |
|  |  |  |  |  |  |  |  |  |
| **Blood** | **Iohexol** | **QCL** | 104.2 | 96.1 | [1.2] |  | 77.8 | [2.0] |
|  |  | **QCH** | 105.9 | 84.7 | [1.2] |  | 86.2 | [2.5] |
|  |  |  |  |  |  |  |  |  |
|  | **Creatinine-d3** | **QCL** | 38.8 | 87.6 | [1.4] |  | 96.6 | [3.9] |
|  |  | **QCH** | 47.2 | 88.4 | [1.9] |  | 93.2 | [4.1] |
|  |  |  |  |  |  |  |  |  |
| **VAMS** | **Iohexol** | **QCL** | 92.3 | 106.8 | [4.7] |  | 106.0 | [5.2] |
|  |  | **QCH** | 83.4 | 103.2 | [2.7] |  | 114.8 | [7.8] |
|  |  |  |  |  |  |  |  |  |
|  | **Creatinine-d3** | **QCL** | 73.7 | 96.0 | [1.6] |  | 107.5 | [6.9] |
|  |  | **QCH** | 89.8 | 96.8 | [1.6] |  | 110.6 | [5.2] |

**Supplementary Table 6** Stability data of iohexol, creatinine-d3 and creatinine in plasma and blood samples after storage for 1 week at 4 °C (n=3). Cat samples are indicated in bold.

|  |  | **Stability expressed as % deviation of nominal value (t0)** | | |
| --- | --- | --- | --- | --- |
|  |  |  | 1 w at 4°C (n=3) | CV (%) |
| Plasma | Iohexol | QCL | 97 | [5.1] |
|  |  | QCH | 100 | [3.7] |
|  |  | **Cat sample 1** | **98** | **[1.5]** |
|  |  | **Cat sample 2** | **103** | **[4.2]** |
|  |  |  |  |  |
|  | Creatinine-d3 | QCL | 93 | [20.6] |
|  |  | QCH | 96 | [4.3] |
|  |  |  |  |  |
|  | Creatinine | QCL | 86 | [8.9] |
|  |  | QCH | 91 | [13.6] |
|  |  | **Cat sample 1** | **94** | **[13.2]** |
|  |  | **Cat sample 2** | **98** | **[8.8]** |
|  |  |  |  |  |
| Blood | Iohexol | QCL | 91 | [3.8] |
|  |  | QCH | 90 | [1.9] |
|  |  | **Cat sample 1** | **89** | **[2.5]** |
|  |  | **Cat sample 2** | **94** | **[13.8]** |
|  |  |  |  |  |
|  | Creatinine-d3 | QCL | 97 | [9.0] |
|  |  | QCH | 96 | [2.5] |
|  |  |  |  |  |
|  | Creatinine | QCL | 95 | [4.9] |
|  |  | QCH | 98 | [5.6] |
|  |  | **Cat sample 1** | **105** | **[19.7]** |
|  |  | **Cat sample 2** | **102** | **[3.8]** |

**Supplementary Table 7** Stability data of iohexol, creatinine-d3 and creatinine in VAMS samples after storage for 1 week at room temperature (RT) and 2 days at 60 °C (n=3). Cat samples are indicated in bold.

|  |  | **Stability expressed as % deviation of nominal value (t0)** | | | | |
| --- | --- | --- | --- | --- | --- | --- |
|  |  |  | 1 w at RT (n=3) | CV (%) | 2 d at 60°C (n=3) | CV (%) |
| VAMS | Iohexol | QCL | 107 | [7.6] | 97 | [4.9] |
|  |  | QCH | 101 | [13.2] | 100 | [6.6] |
|  |  | **Cat sample 1** | **99** | **[6.2]** | **98** | **[7.5]** |
|  |  | **Cat sample 2** | **96** | **[6.6]** | **100** | **[7.8]** |
|  |  |  |  |  |  |  |
|  | Creatinine-d3 | QCL | 98 | [6.4] | 89 | [6.2] |
|  |  | QCH | 94 | [8.4] | 87 | [3.5] |
|  |  |  |  |  |  |  |
|  | Creatinine | QCL | 96 | [7.1] | 136 | [6.6] |
|  |  | QCH | 101 | [6.1] | 128 | [4.1] |
|  |  | **Cat sample 1** | **97** | **[4.5]** | **95** | **[11.4]** |
|  |  | **Cat sample 2** | **93** | **[2.0]** | **87** | **[4.8]** |

**Supplementary Table 8** Long term stability data of iohexol and creatinine determined in a pool of cat blood (from which VAMS were prepared) and plasma with low and high concentrations (n=3) on each study sample analysis day. Concentrations are expressed in µg/mL. The first study sample analysis day is t_0_. For t_1_, t_2_, t_3_, t_4_ and t_5_, the % deviation from t_0_ was determined.

|  |  |  | t_0_ | t_1_ | % deviation from t_0_ | t_2_ | % deviation from t_0_ | t_3_ | % deviation from t_0_ | t_4_ | % deviation from t_0_ | t_5_ | % deviation from t_0_ |
| --- | --- | --- | --- | --- | --- | --- | --- | --- | --- | --- | --- | --- | --- |
| **Plasma** | **Iohexol** | Low | 19.6 | 18.7 | -5 | 19.4 | -2 | 19.7 | 1 | 19.5 | -1 | 20.5 | 4 |
|  |  | High | 194.4 | 190 | -2 | 195.8 | 1 | 200.6 | 3 | 202.6 | 4 | 205.5 | 6 |
|  |  |  |  |  |  |  |  |  |  |  |  |  |  |
|  | **Creatinine** | Low | 25.8 | 27.1 | 5 | 27.8 | 8 | 28.4 | 10 | 28 | 8 | 26.2 | 1 |
|  |  | High | 52.2 | 56.3 | 8 | 58.5 | 12 | 58.1 | 11 | 57.4 | 10 | 52.9 | 1 |
|  |  |  |  |  |  |  |  |  |  |  |  |  |  |
| **Blood** | **Iohexol** | Low | 19.2 | 19.6 | 2 | 18.7 | -3 |  |  |  |  |  |  |
|  |  | High | 129.7 | 120.3 | -7 | 130.1 | 0 |  |  |  |  |  |  |
|  |  |  |  |  |  |  |  |  |  |  |  |  |  |
|  | **Creatinine** | Low | 23.6 | 24.7 | 5 | 25.1 | 6 |  |  |  |  |  |  |
|  |  | High | 44.2 | 44.9 | 2 | 48.4 | 10 |  |  |  |  |  |  |
|  |  |  |  |  |  |  |  |  |  |  |  |  |  |
| **VAMS** | **Iohexol** | Low | 15.9 | 16.7 | 5 | 16.9 | 6 | 18.8 | 18 |  |  |  |  |
|  |  | High | 113.5 | 111.8 | -1 | 109.5 | -4 | 125.7 | 11 |  |  |  |  |
|  |  |  |  |  |  |  |  |  |  |  |  |  |  |
|  | **Creatinine** | Low | 22.3 | 22.7 | 2 | 21.9 | -2 | 26.2 | 17 |  |  |  |  |
|  |  | High | 46.0 | 42.4 | -8 | 41.3 | -10 | 49.9 | 9 |  |  |  |  |

|  |  |  | **Stability expressed as % deviation of nominal value (t0)** | | | | | |
| --- | --- | --- | --- | --- | --- | --- | --- | --- |
|  |  |  | **24h Autosampler Stability (10°C) (n=2)** | **% difference** | **48h Autosampler Stability (10°C) (n=2)** | **% difference** | **Stability for 2 weeks at -20°C (n=2)** | **% difference** |
| Plasma | Iohexol | QCL | 97 | 2% | 97 | 6% | 115 | 2% |
|  |  | QCH | 100 | 0% | 102 | 4% | 101 | 1% |
|  |  | **Cat QC 1** | **102** | **4%** | **103** | **2%** | **103** | **6%** |
|  |  | **Cat QC 2** | **104** | **2%** | **102** | **6%** | **88** | **11%** |
|  |  | **Cat QC 3** | **99** | **2%** | **96** | **4%** | **104** | **8%** |
|  |  | **Cat QC 4** | **101** | **1%** | **100** | **3%** | **96** | **3%** |
|  |  |  |  |  |  |  |  |  |
|  | Creatinine-d3 | QCL | 104 | 4% | 113 | 1% | 107 | 4% |
|  |  | QCH | 101 | 4% | 102 | 2% | 98 | 4% |
|  |  | **Cat QC 1** | **105** | **17%** | **100** | **8%** | **111** | **8%** |
|  |  | **Cat QC 2** | **113** | **12%** | **107** | **11%** | **91** | **17%** |
|  |  | **Cat QC 3** | **106** | **0%** | **105** | **2%** | **86** | **5%** |
|  |  | **Cat QC 4** | **100** | **7%** | **102** | **8%** | **100** | **10%** |
|  |  |  |  |  |  |  |  |  |
|  | Creatinine | QCL | 104 | 33% | 109 | 4% | 96 | 22% |
|  |  | QCH | 126 | 1% | 128 | 9% | 104 | 14% |
|  |  | **Cat QC 1** | **114** | **20%** | **109** | **20%** | **97** | **10%** |
|  |  | **Cat QC 2** | **101** | **3%** | **100** | **4%** | **105** | **5%** |
|  |  | **Cat QC 3** | **112** | **3%** | **104** | **3%** | **68** | **33%** |
|  |  | **Cat QC 4** | **103** | **8%** | **102** | **3%** | **106** | **11%** |

**Supplementary Table 9** Stability data of iohexol, creatinine-d3 and creatinine in blood, plasma and VAMS extracts (n=2). Percentage difference (%) refers to the difference between duplicates divided by the mean of the duplicates. Cat samples are indicated in bold.

**Supplementary Table 9** Stability data of iohexol, creatinine-d3 and creatinine in blood, plasma and VAMS extracts (n=2). Percentage difference (%) refers to the difference between duplicates divided by the mean of the duplicates**.** Cat samples are indicated in bold. (Continued).

|  |  |  | **Stability expressed as % deviation of nominal value (t0)** | | | | | |
| --- | --- | --- | --- | --- | --- | --- | --- | --- |
|  |  |  | **24h Autosampler Stability (10°C) (n=2)** | **% difference** | **48h Autosampler Stability (10°C) (n=2)** | **% difference** | **Stability for 2 weeks at -20°C (n=2)** | **% difference** |
| Blood | Iohexol | QCL | 98 | 8% | 102 | 8% | 93 | 6% |
|  |  | QCH | 105 | 9% | 102 | 14% | 104 | 2% |
|  |  | **Cat QC 1** | **101** | **6%** | **101** | **2%** | **99** | **15%** |
|  |  | **Cat QC 2** | **99** | **2%** | **100** | **0%** | **101** | **4%** |
|  |  | **Cat QC 3** | **101** | **2%** | **100** | **0%** | **98** | **8%** |
|  |  | **Cat QC 4** | **102** | **8%** | **100** | **0%** | **101** | **9%** |
|  |  |  |  |  |  |  |  |  |
|  | Creatinine-d3 | QCL | 96 | 0% | 98 | 0% | 92 | 4% |
|  |  | QCH | 98 | 1% | 99 | 1% | 100 | 4% |
|  |  | **Cat QC 1** | **99** | **2%** | **94** | **0%** | **92** | **4%** |
|  |  | **Cat QC 2** | **96** | **0%** | **100** | **6%** | **103** | **4%** |
|  |  | **Cat QC 3** | **102** | **0%** | **100** | **0%** | **100** | **10%** |
|  |  | **Cat QC 4** | **99** | **2%** | **99** | **1%** | **95** | **1%** |
|  |  |  |  |  |  |  |  |  |
|  | Creatinine | QCL | 103 | 0% | 106 | 6% | 122 | 12% |
|  |  | QCH | 97 | 6% | 100 | 0% | 112 | 27% |
|  |  | **Cat QC 1** | **104** | **3%** | **101** | **3%** | **106** | **2%** |
|  |  | **Cat QC 2** | **102** | **3%** | **97** | **10%** | **104** | **14%** |
|  |  | **Cat QC 3** | **106** | **3%** | **101** | **5%** | **91** | **20%** |
|  |  | **Cat QC 4** | **102** | **9%** | **100** | **0%** | **90** | **15%** |

**Supplementary Table 9** Stability data of iohexol, creatinine-d3 and creatinine in blood, plasma and VAMS extracts (n=2 Percentage difference (%) refers to the difference between duplicates divided by the mean of the duplicates**.** Cat samples are indicated in bold. (Continued).

|  |  |  | **Stability expressed as % deviation of nominal value (t0)** | | | | | |
| --- | --- | --- | --- | --- | --- | --- | --- | --- |
|  |  |  | **24h Autosampler Stability (10°C) (n=2)** | **% difference** | **48h Autosampler Stability (10°C) (n=2)** | **% difference** | **Stability for 2 weeks at -20°C (n=2)** | **% difference** |
| VAMS | Iohexol | QCL | 102 | 0% | 97 | 6% | 97 | 7% |
|  |  | QCH | 99 | 2% | 103 | 7% | 95 | 1% |
|  |  | **Cat QC 1** | **94** | **9%** | **97** | **5%** | **97** | **2%** |
|  |  | **Cat QC 2** | **101** | **3%** | **104** | **2%** | **95** | **1%** |
|  |  | **Cat QC 3** | **98** | **7%** | **97** | **7%** | **96** | **4%** |
|  |  | **Cat QC 4** | **99** | **1%** | **102** | **6%** | **98** | **2%** |
|  |  |  |  |  |  |  |  |  |
|  | Creatinine-d3 | QCL | 99 | 6% | 101 | 6% | 102 | 8% |
|  |  | QCH | 97 | 0% | 100 | 1% | 99 | 4% |
|  |  | **Cat QC 1** | **95** | **2%** | **96** | **0%** | **96** | **8%** |
|  |  | **Cat QC 2** | **98** | **3%** | **102** | **6%** | **102** | **7%** |
|  |  | **Cat QC 3** | **95** | **6%** | **104** | **4%** | **95** | **6%** |
|  |  | **Cat QC 4** | **97** | **4%** | **99** | **5%** | **103** | **4%** |
|  |  |  |  |  |  |  |  |  |
|  | Creatinine | QCL | 97 | 0% | 93 | 7% | 85 | 8% |
|  |  | QCH | 91 | 6% | 86 | 17% | 79 | 5% |
|  |  | **Cat QC 1** | **107** | **2%** | **101** | **2%** | **95** | **11%** |
|  |  | **Cat QC 2** | **96** | **12%** | **100** | **0%** | **95** | **3%** |
|  |  | **Cat QC 3** | **96** | **9%** | **97** | **2%** | **104** | **19%** |
|  |  | **Cat QC 4** | **98** | **4%** | **100** | **0%** | **102** | **0%** |

**Supplementary Table 10** Characteristics of the patient population. In total 51 different cats were included of which 13 were sampled twice with one year in between. In the clinical validation study 23 cats were included. In the application study 40 cats were included.

| **Race, n**  **Age (years) median (range)**  **Gender (males/females)** | European Shorthair (n=41)  British Shorthair (n=4)  Maine Coon (n=2)  Ragdoll (n=2)  Somali (n=1)  Devon Rex (n=1)  13 (6-18)  23 M / 29 F |
| --- | --- |
